# Supplementary material for: Integrated and DC-powered superconducting microcomb
Source: Nat Commun. 2024 May 13;15:4009. doi: 10.1038/s41467-024-48224-1 (PMC11091051; doi:10.1038/s41467-024-48224-1)
Supplement: Supplementary file 1 — Supplementary Information [file 41467_2024_48224_MOESM1_ESM.pdf]

Supplementary Information for

Integrated and DC-powered superconducting microcomb

Chen-Guang Wang[1,2,3], Wuyue Xu[1,2,3], Chong Li[1,2,3], Lili Shi[1], Junliang Jiang[1], Tingting Guo[1], Wen-Cheng Yue[1,2,3], Tianyu Li[1,2,3], Ping Zhang[1], Yang-Yang Lyu[1,2], Jiazheng Pan[2], Xiuhao Deng[4,5], Ying Dong[6], Xuecou Tu[1,5], Sining Dong[1,3], Chunhai Cao[1], Labao Zhang[1,5], Xiaoqing Jia[1,5], Guozhu Sun[1,5], Lin Kang[1,5], Jian Chen[1,2], Yong-Lei Wang[1,2,3,*], Huabing Wang[1,2,*], Peiheng Wu[1,2,*]

[1] *Research Institute of Superconductor Electronics, School of Electronic Science and Engineering, Nanjing University, Nanjing, China*
[2] *Purple Mountain Laboratories, Nanjing, China*
[3] *National Key Laboratory of Spintronics, Nanjing University, SuZhou, China*
[4] *Shenzhen Institute for Quantum Science and Engineering, Southern University of Science and Technology, Shenzhen, China*
[5] *Hefei National Laboratory, Hefei, China*
[6] *College of Metrology Measurement and Instrument, China Jiliang University, Hangzhou, China*

* Correspondence to:

yongleiwang@nju.edu.cn;

hbwang@nju.edu.cn;

phwu@nju.edu.cn

The PDF file includes:

Supplementary Figures 1 to 13
Supplementary Table 1

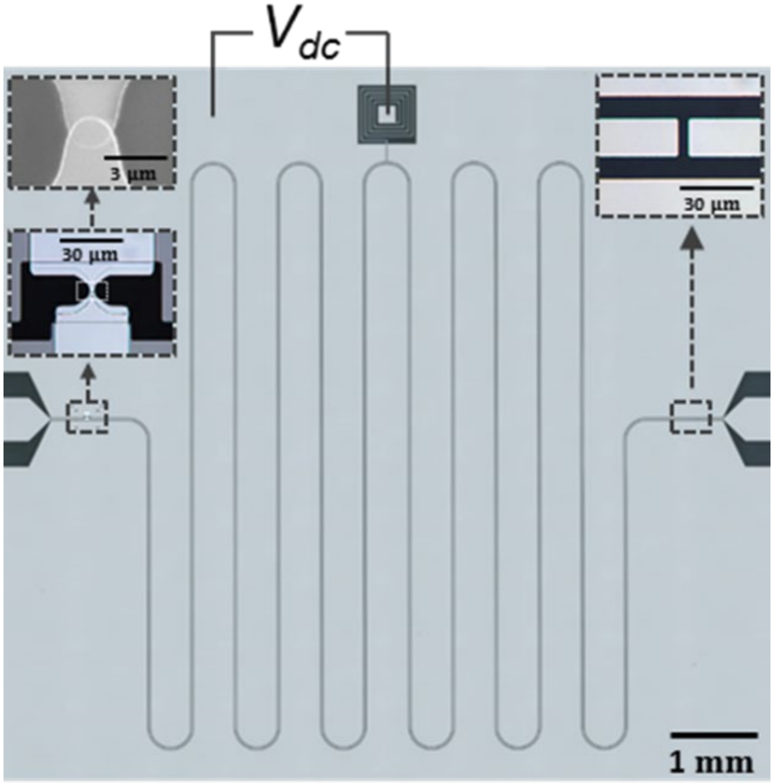

Supplementary Figure 1 | Sample image. The Josephson junction is strongly coupled to a half-wave superconducting CPW resonator. The length of the center conductor of the resonator is around 10 mm, corresponding to the fundamental frequency of 750 MHz. The size of the Josephson junction is approximately 4 μm^2.

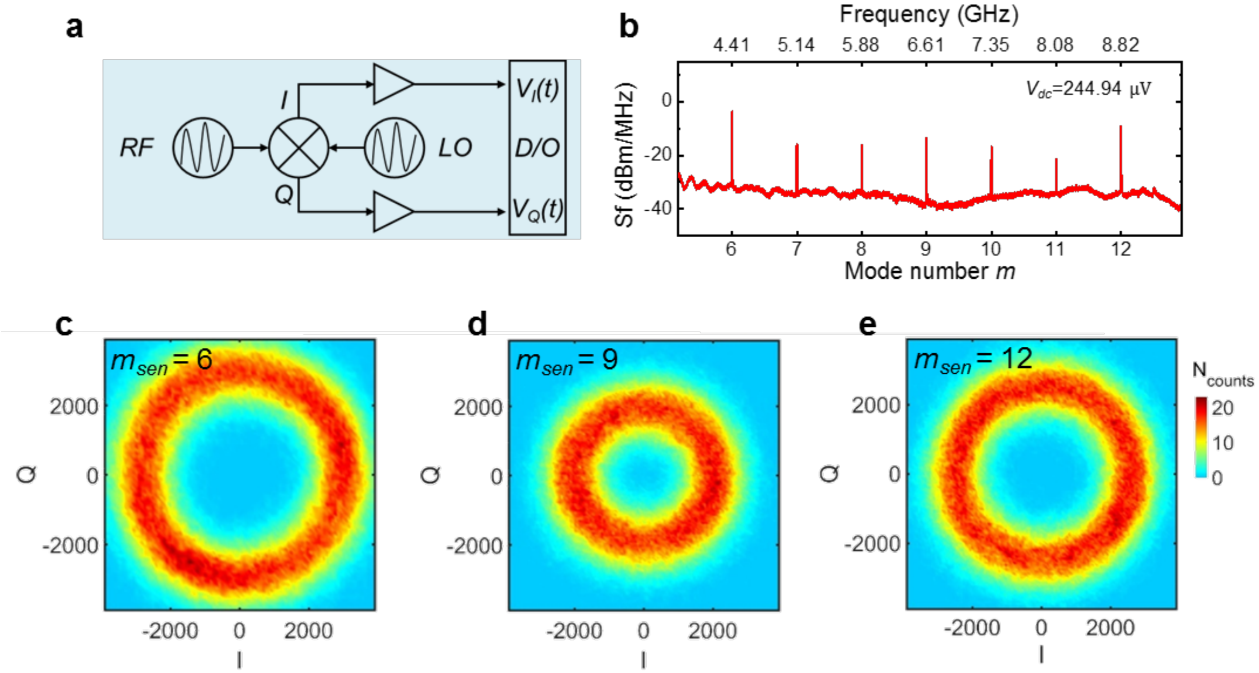

Supplementary Figure 2 | Coherent emission of each individual comb mode. (**a**) The schematic diagram of the heterodyne detection technique. The emitted comb signal is demodulated by a local reference tone to yield the in-phase (I) and quadrature-phase (O) components. The frequency of the reference tone is, in turn, detuned from the sensing modes, f_6, f_9 and f_{12}, respectively, by 2MHz. (**b**) Comb spectrum. (**c**-**e**) Two-dimensional probability distributions for m_{sen}=6 (c), 9 (d) and 12 (e), respectively. Each IQ histogram contains 5×10^4 sampling points.

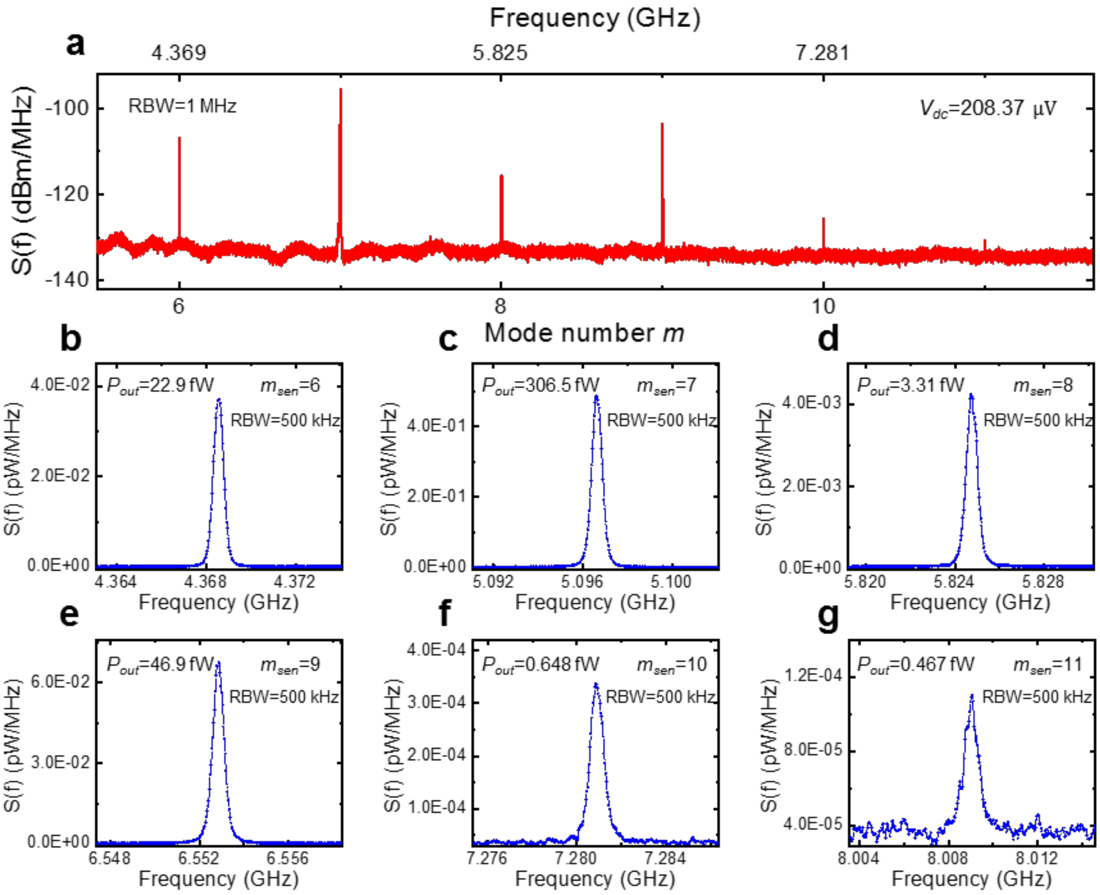

Supplementary Figure 3 | Comb emission power. (**a**) A free-running comb spectrum. (**b-g**) Zoomed-in comb spectra with calculated powers for each mode.

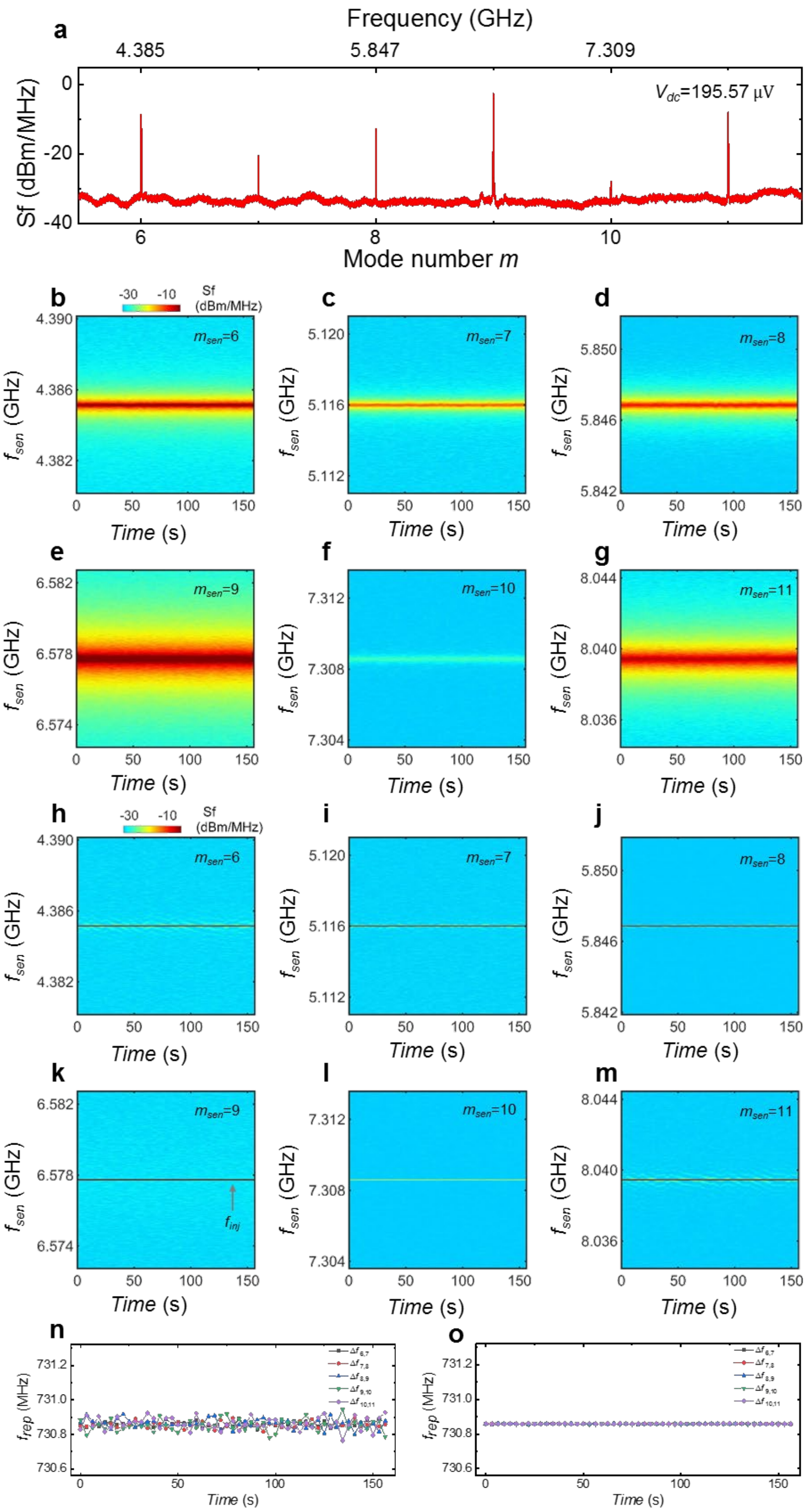

Supplementary Figure 4 | Stability of frequency combs. (**a**) a comb spectrum. (**b-g**) Time dependence of the free-running comb spectrum maps. (**h-m**) Time dependence of the injection-locked comb spectrum maps. (**n** and **o**) Time dependence of line spacing for the free-running comb (n) and injection-locked comb (o).

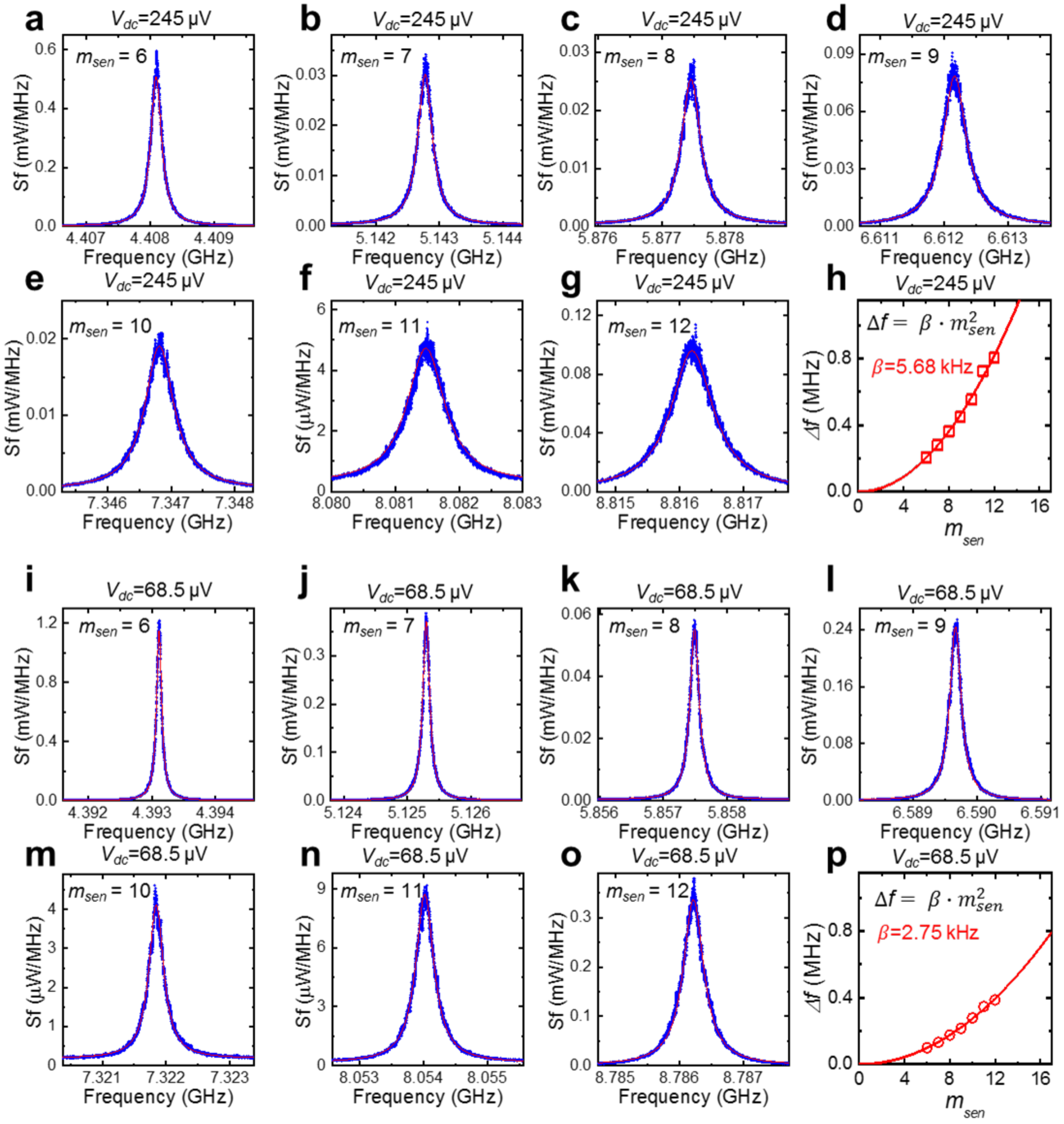

Supplementary Figure 5 | Mode number dependence of linewidth. The mode spectra are fitted by Lorentzian functions and the linewidths at half maximum are extracted. (**a-g**) Spectra of various modes obtained under V_{dc}=245 μV. The red lines are fittings using Lorentzian functions. (**h**) Extracted line width Δf from fittings in (a-g). (**i-p**) The results obtained under V_{dc}=68.5 μV from the same device. The linewidth of the Josephson comb increases quadratically with the mode number. It also varies with V_{dc}.

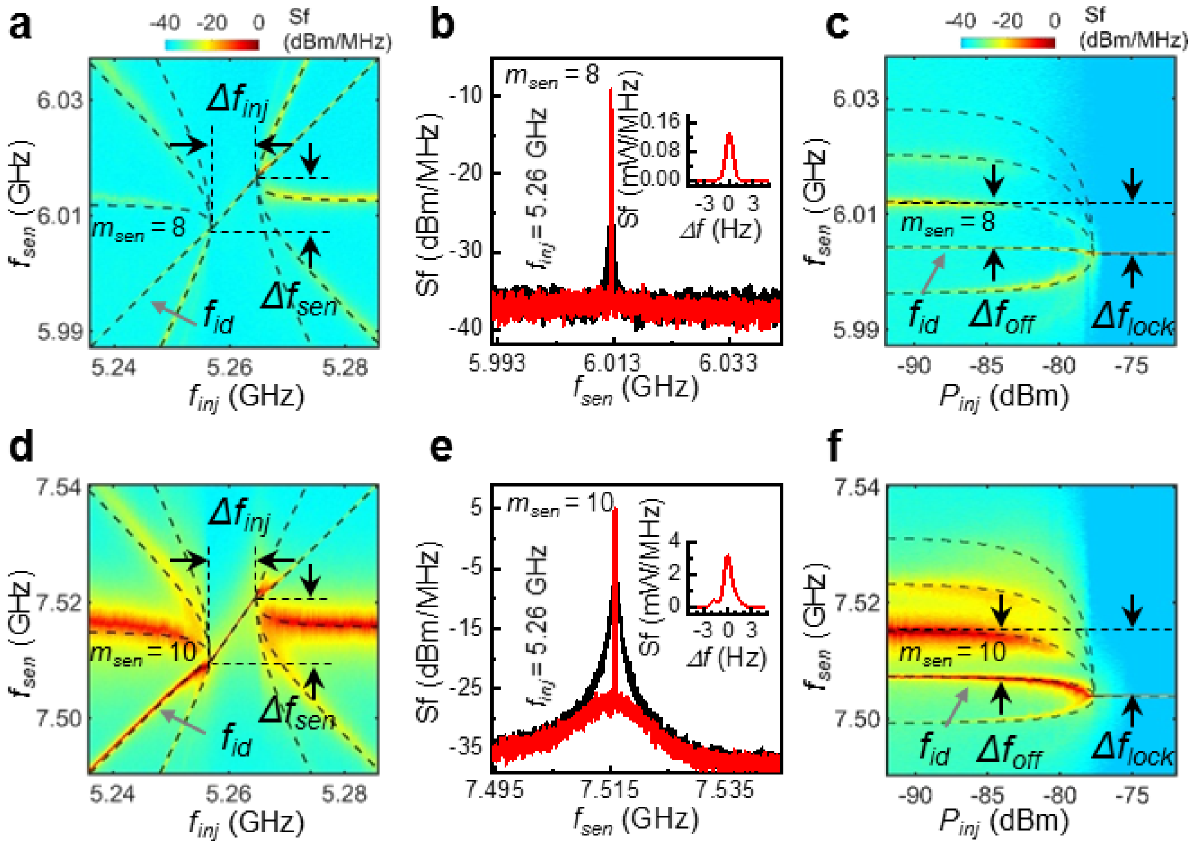

Supplementary Figure 6 | Coherent injection locking data of the other sensing modes in Fig. 3. (**a-c**) Results for m_{sen}=8. (**d-f**) Results for m_{sen}=10.

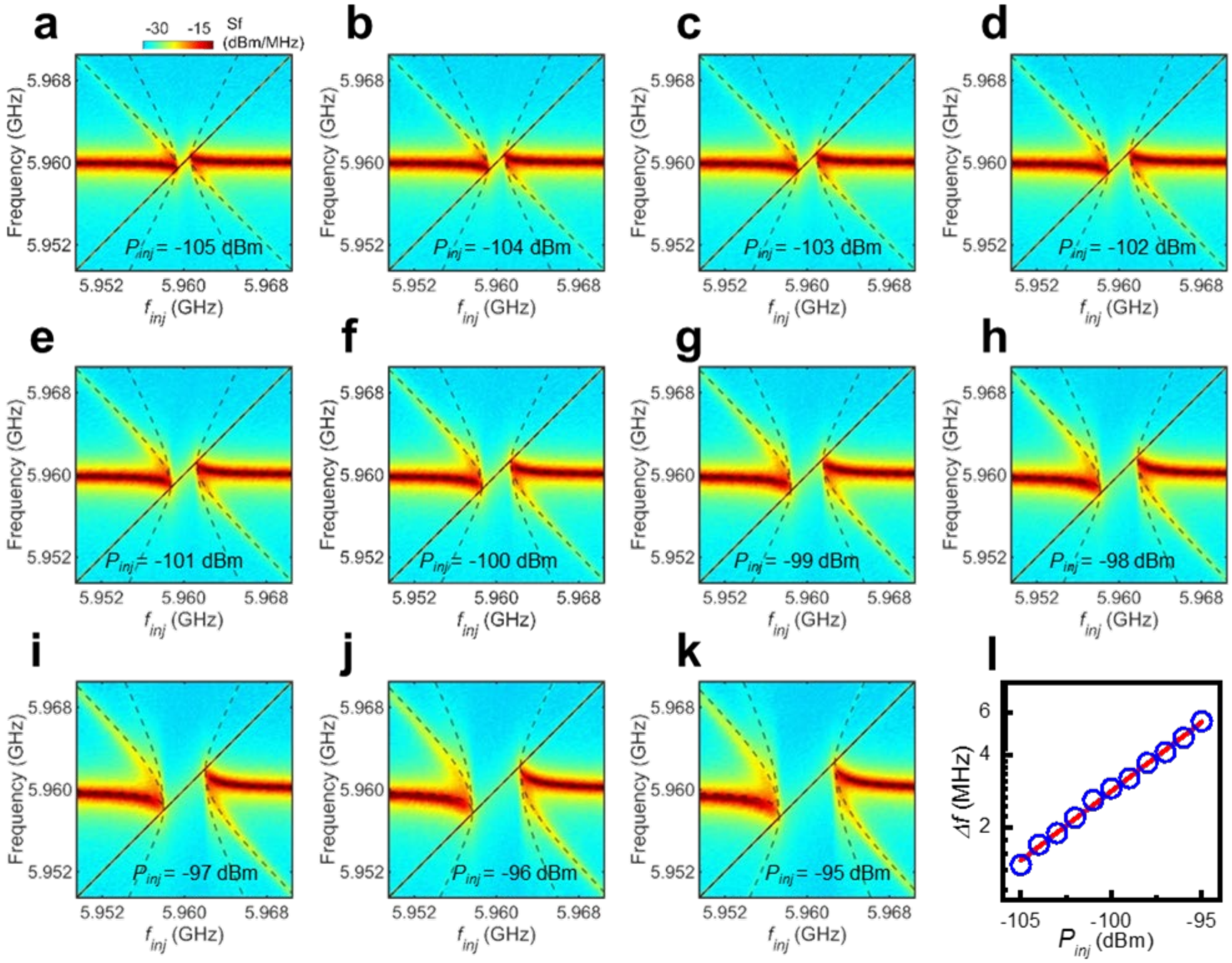

Supplementary Figure 7 | Injection-locking range Δf_{inj} as a function of injection power P_{inj} and measured at the same sensing tone. (**a-k**) Power spectral density of injection locking under different injection powers from -105 dBm to -95 dBm. (**l**) Extracted frequency range Δf_{inj} from (a-k) as a function of injection power P_{inj}. The result is in good agreement with the fitting (red line) to the Adler's theory.

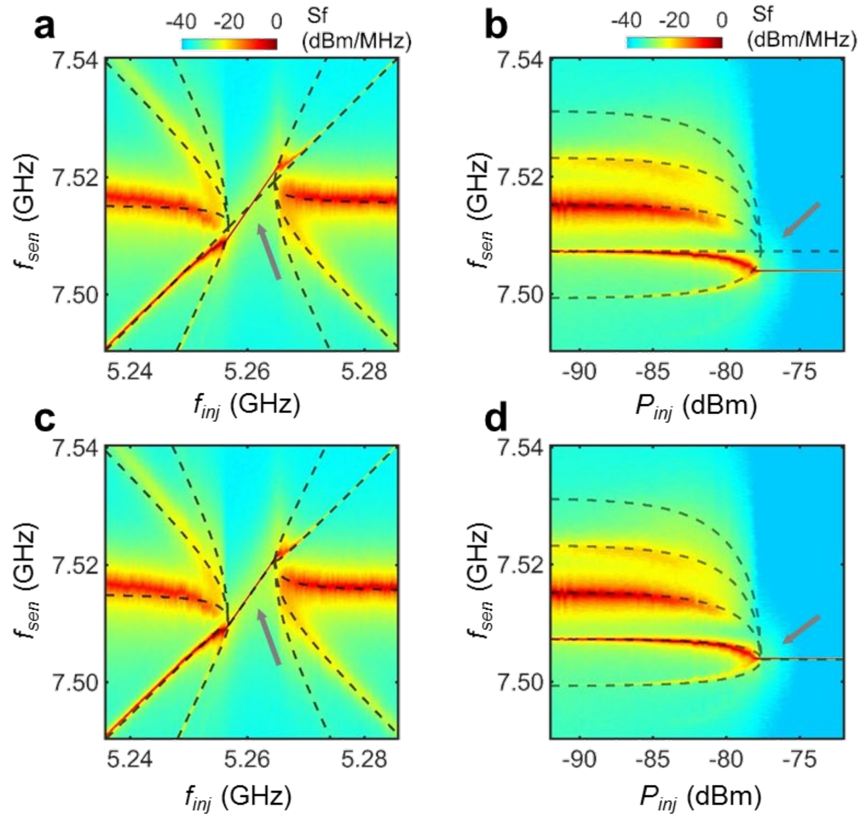

Supplementary Figure 8 | The failure of Alder's theory on coherence injection locking. (a-b) Fittings to Alder's theory for the injection-locking spectra of 'm_{inj}=7, m_{sen}=10' as a function of f_{inj} (a) and P_{inj} (b). Clear deviations in the locking range (marked by arrows) indicate the failure of Alder's theory on the coherence injection locking for $m_{sen} \neq m_{inj}$. **(c-d)** Fittings to the extended Alder's equation (2) match perfectly with the experimental data.

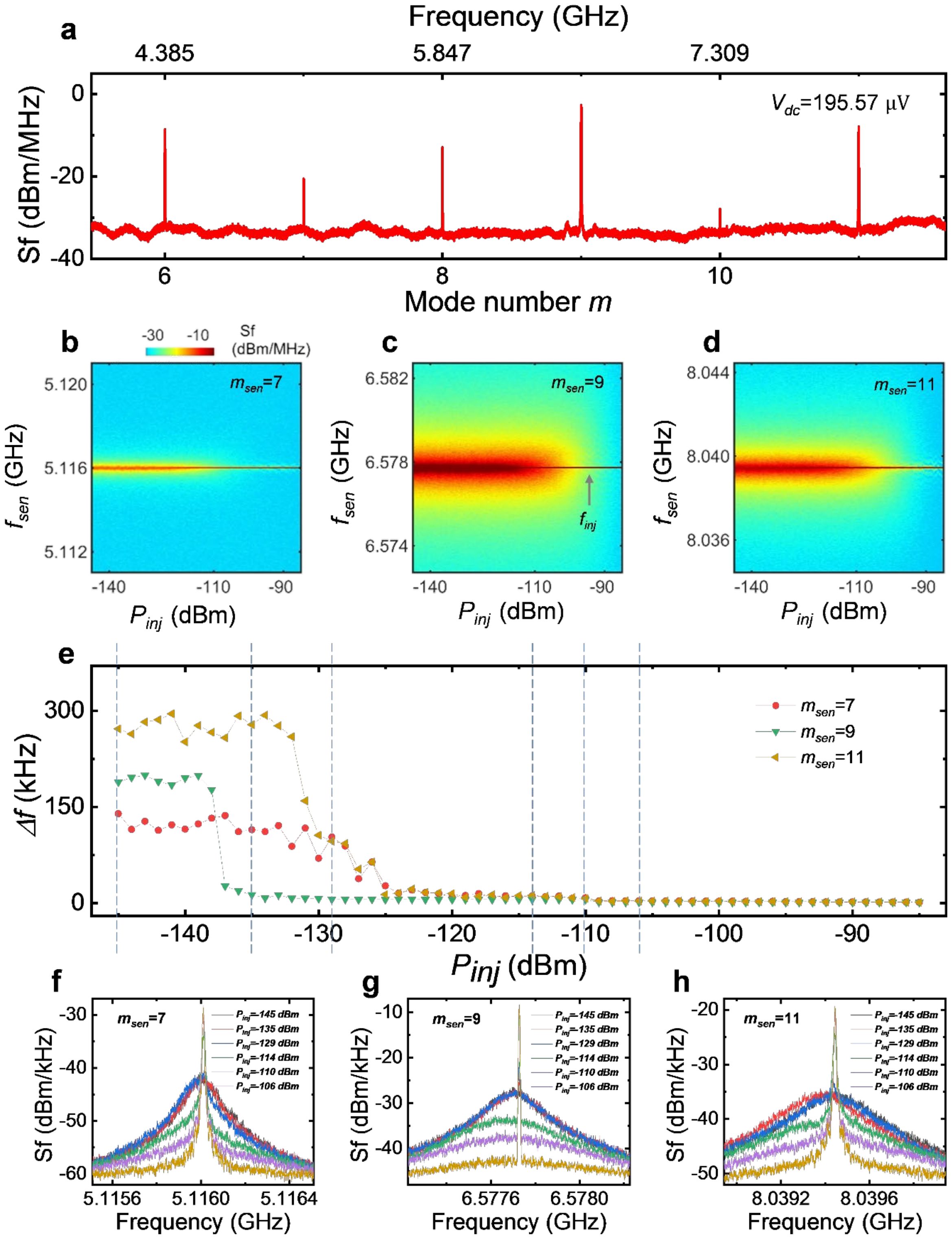

Supplementary Figure 9 | Dependence of linewidth on injection power. (**a**) Spectrum of the free running comb. (**b-d**) Spectra maps obtained by varying injection power (P_{inj}) under m_{inj}=9 for m_{sen}=7, 9, 11, respectively. (**e**) Extracted linewidths plotted as a function of P_{inj}. (**f-h**) Spectrum lines with selected P_{inj} values.

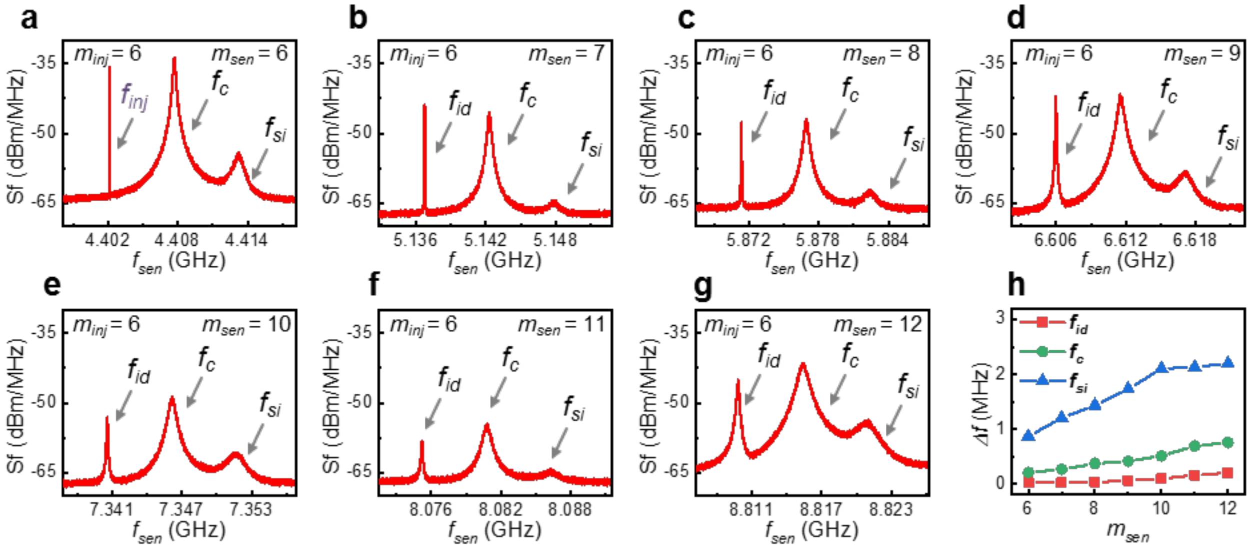

Supplementary Figure 10 | Induced-injection tones. (**a**-**g**) Power spectral density at different values of m_{sen} for an off-resonance injection. Emissions of induced-injection tones f_{id} emerge at $m_{sen} \neq m_{inj}$ (b-g). (**h**) The linewidths of f_{id} (red), the comb emission tone f_c (green) and side-band/harmonic emission tone f_{si} (blue) as a function of m_{sen}. The linewidth of f_{id} is gradually broadening with m_{sen}, and is broader than that of injection tone f_{inj} (a), but much narrower than those of f_c and f_{si} (h).

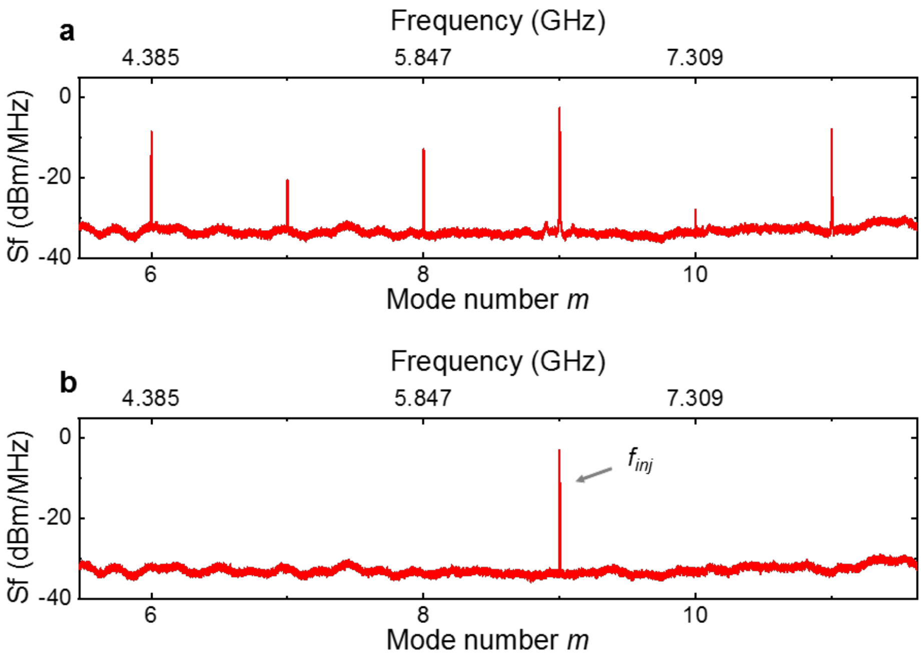

Supplementary Figure 11 | Comparison between microwave injection with and without comb emission. (**a**) Spectrum with injection tone m_{inj}=9 (6.578 GHz) and at V_{dc}=195.57 µV (with comb emission). (**b**) Spectrum with the same injection tone and at V_{dc} = 0 V (without comb emission).

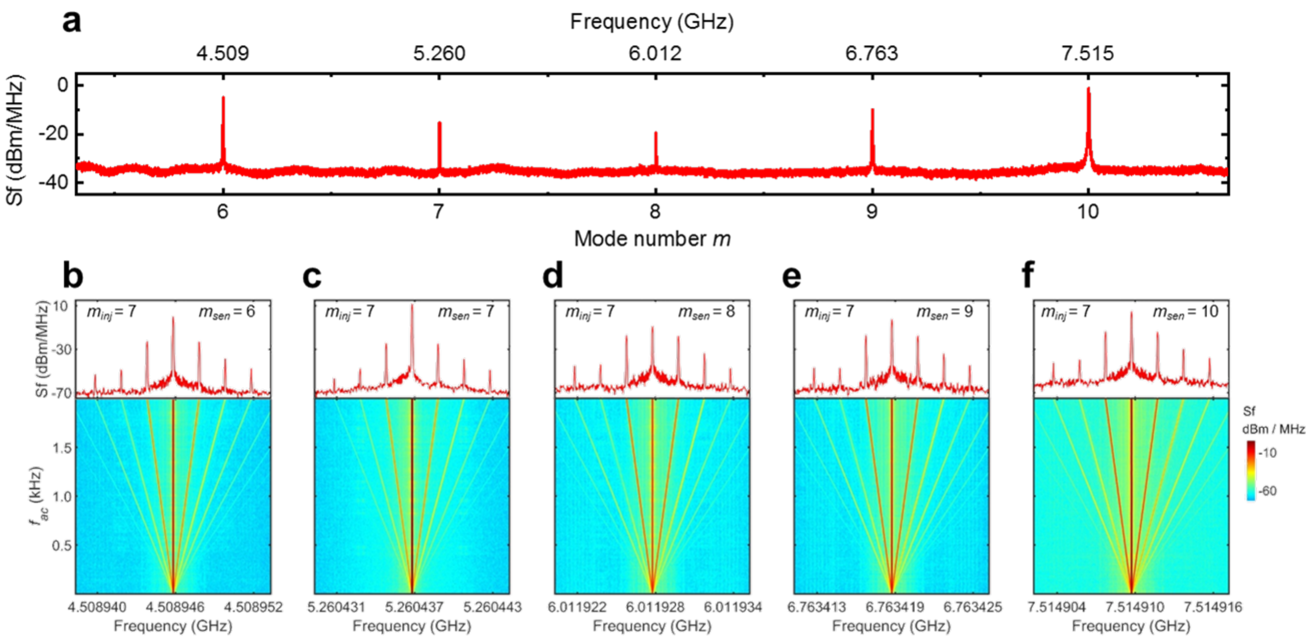

Supplementary Figure 12 | Coherent subcombs. (**a**) Spectrum of an injection-locked superconducting microcomb obtained at V_{dc} = 237.6 μV. The parameters of injection-locking are the same to those in Fig.3. (**b**-**f**) Synchronized subcombs generated by four-wave mixing, in which we apply a small AC excitation of frequency f_{ac} to the DC bias applied to the Josephson junction. The amplitude of the applied AC excitation is one percent of the DC voltage bias.

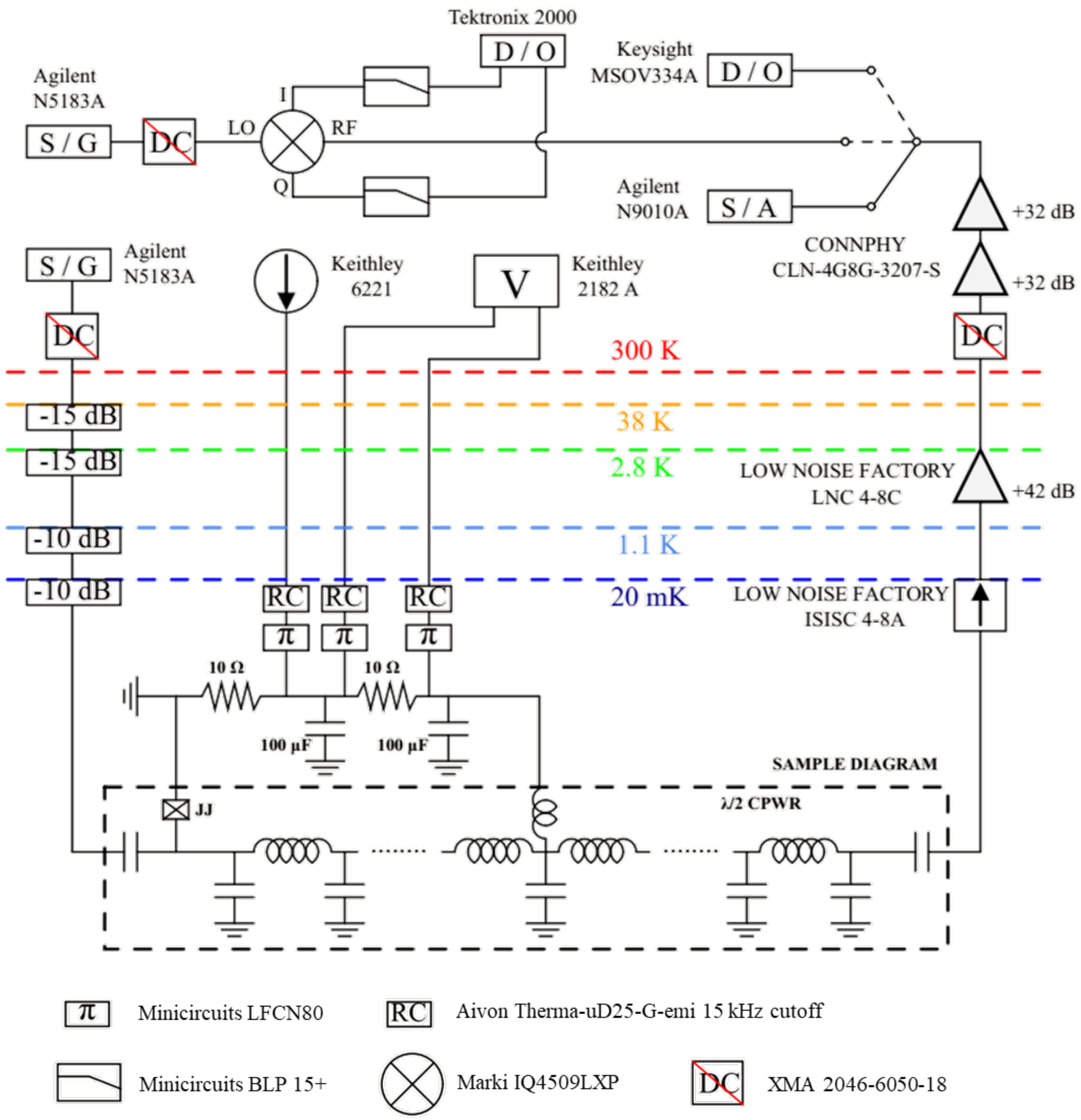

Supplementary Figure 13 | Measurement circuit schematic. The device is mounted in a dilution refrigerator with a base temperature of 20 mK. The bias voltage is supplied by an on-chip voltage divider circuit consisting of a 10 Ω shunt resistor and a 10 Ω reference resistor. All low frequency lines are heavily filtered using multi-pole RC and π low-pass filters. The device's output signal is amplified by an amplifier chain consisting of a cryogenic amplifier (+42 dB) and two room-temperature amplifiers (+32 dB). The injection signal to the resonator is attenuated by a low-temperature attenuation chain (-50 dB) to assure that the thermal contribution of photons to the cavity is negligible.

Supplementary Table 1 | Device and Experimental Parameters

Sample #	Resonator type	Resonator material	Size of JJ (μm^2)	V_{dc} (μV)	I_D (μA)	Figure #
#1	CPW (750MHz)	Nb 120 nm thick	4.85	/	/	1b
				43.00	2.05	1c, 1d
				237.60	2.36	3, S6, S10, S12
#2	CPW (750MHz)	Ta (α-phase) 180 nm thick	5.08	83.28	1.68	2
				36.39	1.43	4
				244.94	1.99	S2, S8, S5a-S5h
				60.50	1.87	S5i-S5p
#3	CPW (750MHz)	Ta (α-phase) 180 nm thick	6.99	110.51	2.72	S7
				208.37	2.75	S3
#4	CPW (750MHz)	Ta (α-phase) 180 nm thick	6.34	195.57	1.96	S11, S9, S4
